# The impact of the implementation of work hour requirements on residents' career satisfaction, attitudes and emotions

**DOI:** 10.1186/1472-6920-6-53

**Published:** 2006-10-17

**Authors:** Dongseok Choi, Jamie Dickey, Kristen Wessel, Donald E Girard

**Affiliations:** 1 Department of Public Health and Preventive Medicine, School of Medicine, Oregon Health & Science University, Portland, OR, USA; 2 Department of Psychiatry, School of Medicine, Oregon Health & Science University, Portland, OR, USA; 3 Graduate Medical Education, School of Medicine, Oregon Health & Science University, Portland, OR, USA; 4 Department of Anesthesiology, School of Medicine, Oregon Health & Science University, Portland, OR, USA

## Abstract

**Background:**

To assess the impact of work hours' limitations required by the Accreditation Council for Graduate Medical Education (ACGME) on residents' career satisfaction, emotions and attitudes.

**Methods:**

A validated survey instrument was used to assess residents' levels of career satisfaction, emotions and attitudes before and after the ACGME duty hour requirements were implemented. The "pre" implementation survey was distributed in December 2002 and the "post" implementation one in December 2004. Only the latter included work-hour related questions.

**Results:**

The response rates were 56% for the 2002 and 72% for the 2004 surveys respectively. Although career satisfaction remained unchanged, numerous changes occurred in both emotions and attitudes. Compared to those residents who did not violate work-hour requirements, those who did were significantly more negative in attitudes and emotions.

**Conclusion:**

With the implementation of the ACGME work hour limitations, the training experience became more negative for those residents who violated the work hour limits and had a small positive impact on those who did not violate them. Graduate medical education leaders must innovate to make the experiences for selected residents improved and still maintain compliance with the work hour requirements.

## Background

The Accreditation Council for Graduate Medical Education (ACGME) implemented residents' work hour limitations in July 2003 to improve patient safety. Another important potential impact of more limited work hours is change in residents' career satisfaction. Virtually all studies of residents' career satisfaction, emotions and attitudes at mid year have demonstrated negative findings, many of which have been reported in studies from this institution and from collaborations with others [1-14]. Indeed, many authors have proposed that excessive work hours have been at least to a degree responsible for those negative findings [3,5,6,12-15]. Thus one would intuitively think that fewer hours of duty might result in more favorable feelings about career satisfaction, emotions and attitudes.

This study utilized a survey instrument, previously validated to demonstrate changes in attitudes and emotions with the Profile of Mood States [10], to evaluate satisfaction with career choice and emotional states among all residents and all faculty in one large academic medical center. Since 2002, the division of graduate medical education has surveyed all residents at Oregon Health & Science University utilizing the instrument to assess residents' levels of career satisfaction, attitudes and emotions. In July 2003, the institution implemented requirements to assure that residents complied with the ACGME duty hour limitations. To date there have been a small number of studies comparing various factors before and after intervention of the ACGME work hour limitations. None have included the entire resident cohort or provided multiple comparisons [16, 17, 18, 19, 20]. This study reports the results of a comparison of career satisfaction, emotional and attitudinal changes and self-reports of work hours among all residents between the survey in the 2002–03 academic year and the survey in the 2004–05 academic year, one and one-half years after implementation of the 80-hour work hour regulations.

## Method

The multiple question survey was distributed to all residents at Oregon Health & Science University (OHSU) in December 2002 [21] and December 2004, before and after the implementation of the ACGME duty hour requirements, respectively. Specific questions compared included responses to levels of career satisfaction (5-point Likert scale), 31 questions (Y/N) about positive (14) and negative (17) aspects of training, and 15 responses to questions about positive or negative emotional states (5-point Likert scale). Both surveys took place at mid-year, a time that typically represents the peak period of negative feelings and attitudes among residents about their career choices. The 2004 survey included duty hours reporting averaged over four weeks but did not include the other ACGME requirements. Duty hours averaged over a four-week period was chosen because it reflects most closely total working hours and it is the first question in the ACGME residents' survey.

The sets of survey data for the two cohorts were explored by using descriptive statistics. They were compared for pre and post intervention analysis. For further evaluation of the effect of the work hours' requirements, the subgroups of programs where there were reported violations were compared. For the pairwise comparisons between the two groups, the Fisher's exact test was used for both 2 × 2 and 2 × 5 contingency tables.

## Results

In 2002, 327 out of 581 residents (56%) completed the survey and 450 out of 625 (72%) in 2004. The distribution of demographics and years in training of the participants were very similar to those of all residents in the same year, respectively (data not shown). Residents were graduates of most of the US allopathic medical schools, a small number of osteopathic medical schools, and a few international medical schools. The majority of residents came from communities outside of Oregon.

In order to evaluate carefully the relationship between duty hours and emotions and attitudes, the entire post intervention cohort was first compared to the entire pre intervention cohort. The first column in Table 1 shows the p-values from the Fisher's exact test for all emotional descriptors. There was no difference in career satisfaction between the groups. Among the 15 emotional descriptors, the post intervention group was significantly more relieved (p = 0.01) and bored (p = 0.04) than the pre intervention group.

The first column in Table 2 displays the p-values from the Fisher's exact test for the 31 positive and negative experiences with the corresponding proportions of "yes" responses. Compared to the pre intervention group, a higher proportion of the post intervention group experienced "feeling more competent about patient care" (p < 0.005) and at the same time were more concerned about "seeing patients die", (p < 0.005), "dealing with patients with self destructive diseases" (p = 0.02), "dealing with varying opinions of consultants" (p = 0.03) and "getting too little sleep" (p = 0.01). Overall, the differences between the total cohorts before and after the implementation of 80 hr requirement were less significant than hypothesized.

For further evaluation of the impact of the implementation of the duty hour requirements, residency programs were selected from the post intervention group where duty hour violations had been self-reported. Those post intervention residents were further divided into two groups, self reported violators (PostV, 57 residents) and non-violators (PostNV, 230 residents). A third group was identified (PreM, 205 residents) who represented residents from the pre intervention programs where there was any violation reported in the post intervention survey. Then three groups, PostV, PostNV and PreM were compared to one another. The fourth, fifth and sixth columns in Table 1 show the p-values from the Fisher's exact test for the pairwise comparisons of emotional states for PostV vs. PreM, PostNV vs. PreM, and PostV vs. PostNV, respectively. The post intervention violators (PostV) showed significantly more fatigue compared to both post intervention non-violators (PostNV) and pre intervention matched group (PreM). The main differences in emotional descriptors among the three groups are the negative emotions as one can easily see in Table 1. In summary, the post intervention violators (PostV) reported stronger feelings for numerous negative emotions compared to those of the pre intervention matched group (PreM) and the post intervention non violators (PostNV). In contrast, the post intervention non-violators (PostNV) showed little difference when compared to pre intervention matched group (PreM). The significant differences between the post intervention violators (PostV) and the pre intervention matched residents (PreM) and the post intervention non violators (PostNV) are consistent with the impact of the implementation of 80-hour requirement.

With respect to positive and negative aspects of training experiences among these three groups, all show moderate differences among one another. (See column 4 to 6 in Table 2.) In particular, the post intervention violators (PostV) reported higher levels of negative experiences than the post intervention non violators (PostNV) in 5 of 17 negatives training experience questions, including "meeting the unrealistic expectations of faculty" (p = 0.02), "getting too little sleep" (p = 0.01) and "having to see too many patients in too little time" (p = 0.01).

## Discussions and Conclusion

This study utilized a survey instrument to evaluate the impact of duty hours' implementation on residents' career satisfaction, emotions and attitudes. In survey data, a response rate of 50% – 60% is considered acceptable for analysis and reporting [22]. Our response rates were in that range or higher, 56% and 72%, respectively. In addition, the distribution of demographics of respondents at each survey was similar to that of the entire resident cohort at the time of the surveys. Hence, the results are representative of the entire cohort of residents of each survey time. Although there were modest improvements in these factors for the cohort after implementation of duty hour restrictions, there were notably negative changes in emotions and attitudes for selected groups, those who violated the requirements. It is likely that both the modest positive changes for the overall cohort and the notably negative changes for selected groups are both related to the impact of the restricted work hours, since almost no other changes occurred; i.e., residents' demography, salaries and benefits, clinical rotations, support staff, facilities, faculty, and institutional relationships. Between the first and second surveys, ACGME accredited programs increased from 57 to 60. Total residents (including fellows) increased from 581 to 625. Eighteen programs were primary specialties at the time of both surveys. Thirty-nine programs were subspecialty fellowships at the time of the first survey and increased to 42 at the time of the second one. Approximately 75% or trainees were in primary specialty programs at the time of both surveys.

One of the most interesting and unexpected findings of the study is related to the negative changes for those residents who violated the new requirements. Those residents held significantly more negative emotional states and were significantly more negative about the experience. It may be that those residents feel worse about their experiences for several clear reasons, most importantly because of loss of control. In many programs the onus for leaving a duty session is the responsibility of the resident, who has to make the closure of the duty period known. The individual cannot stay "till the work is done", a major change in the traditional paradigm. Not only is the resident uncomfortable giving over unfinished work to others, including the faculty, but such closure engenders negative feelings by those who are left to complete the task; i.e., faculty and senior, often chief residents. For some, leaving at a required time steals them from potentially interesting educational activities that are just beginning in which they cannot participate because their work hours have expired. Whatever the reasons, there is a significant number of trainees whose emotional states and attitudes have worsened after implementation of the work hour requirements and program directors and leaders must be aware of these changes and take action to improve them.

Fewer work hours have been reported to be associated with better attitudes and more favorable emotions. In a prospective, unpublished report comparing emotions and attitudes between U.S. and Australian PGY-1 residents over a year period, the Australian residents had many fewer negative emotions and experiences than did otherwise similar U.S. residents, and the only meaningful difference among variables was a work hour average of 56 hours for the Australians and more than 100 hours for the U.S. residents.

The significant changes in how the residency experiences are perceived are worthy of comment. The average resident after the implementation of work hours limitations reports less fatigue, more sleep, more free time and time with family. That individual also seems to feel less competent about patient care, but at the same time, less anxious about skills' development, less burdened by seeing the same kinds of patients over and over and less criticized by staff. All these positive changes are consistent with the implementation of reduction in work hours.

Several limitations to the study could be cited. One perceived limitation is that this study is a report from a single institution, albeit a relatively large one. We do not believe that this fact lessens the generalizability of the results, since virtually all previously reported studies from this institution have shown consistent patterns [1,9,10], even when cohorts from other institutions were studied simultaneously. Self-reporting of duty hours has some inherent validity concerns. However, the vast majority of studies of similar themes and reports from the ACGME have used self reported data [18, 19, 20, 23, 24], and our institutional data compare favorably with the results of ACGME residents' surveys and ACGME site visit outcomes. Because the requirement did not exist at that time of the first survey, it contained no questions about duty hours. Thus, direct comparison of work hours between the two periods is not available. However the Division of Graduate Medical Education has information from its internal reviews, electronic monitoring systems, ACGME resident surveys and site visits about all programs' changes in duty hours over the study period. In July 2003, with initial implementation of the duty hour requirements, all 54 ACGME accredited programs were surveyed to identify program frequencies of non-compliance with the requirement for 80 hours per week averaged over a 4-week period. Reporting rates ranged from 18–100%. From that information compliance data were calculated. Twenty-two programs, representing approximately half of all residents, were considered at high risk for non-compliance because one or more resident reported excess working hours. Between July 2003 and the time of the December 2004 survey, reports of violations were significantly reduced although not eliminated. At that time six programs, representing approximately 33% of all residents, continued to be out of compliance by at least one respondent.

We believe that work hour relief is important and should be a contribution to improved emotions and perceptions of experiences with residency training. And, for most residents that seems to be the case. In those programs where implementation of these requirements results in more negative changes, program directors and department leaders must innovate. Part of that process must include that the faculty fully support the residents' departure at the appropriate time, that there be appropriate resources to "transition" patients' care, and that there be alternative experiences for those residents who "miss out on opportunities" because of work hour limits. The important lesson from this study is that work hour limits seem to improve emotional and attitudinal factors for most, but not all residents. For the latter groups, innovation is required to improve the experiences.

## Competing interests

The author(s) declare that they have no competing interests.

## Authors' contributions

DC performed statistical analyses, interpreted the results and drafted the manuscript; JD designed the survey and revised the draft critically, KW revised the survey questionnaire, revised the draft critically; DEG supervised the whole project, designed the survey and drafted the manuscript. All authors read and approved the final manuscript.

## Pre-publication history

The pre-publication history for this paper can be accessed here:



## Figures and Tables

**Table 1:** The changes in residents' career satisfaction, attitudes and emotional states before and after implementation of the duty hour requirements.

		Selected group comparison		
	The entire cohort Post:Pre	PostV:PreM	PostNV:PreM	PostV:PostNV
Satisfaction	0.96	0.41	0.96	0.41
Competent	0.67	0.21	0.95	0.29
Excited	0.72	0.75	0.47	0.72
Important	0.50	0.69	0.82	0.51
Relieved	***0.01***	0.09	***0.04***	0.65
Useful	0.10	***0.05***	0.80	0.13

Angry	0.17	***0.02***	0.36	0.14
Anxious	0.73	0.27	0.39	0.20
Bored	***0.04***	***0.01***	0.40	0.11
Depressed	0.12	***0.00***	0.33	***0.01***
Defeated	0.06	***0.00***	***0.03***	0.06
Fatigued	0.66	***0.00***	0.19	***0.00***
Inferior	0.67	***0.00***	0.83	***0.00***
Lonely	0.29	***0.00***	0.52	***0.00***
Nervous	0.23	0.57	0.32	0.10
Overwhelmed	0.50	0.12	0.47	0.40

The p-values were computed by the Fisher's exact test. The p-values in italics are significant at 5% level.

• Selected group: residents from programs where there were violations.

• PostV: violators of the selected group in the survey after implementation.

• PostNV: non-violators of the selected group in the survey after implementation.

• PreM: pre intervention residents in those programs where there were violations in the post intervention survey.

**Table 2:** The changes in residents' experience in positive and negative aspects of training.

	The entire cohort			Selected group comparison					
	p-value	Proportion		p-value			Proportion		
	Post:Pre	Post	Pre	PostV:PreM	PostNV:PreM	PostV:PostNV	PostV	PostNV	PreM
Seeing sick people get better because of my action	0.26	0.84	0.87	0.22	0.36	0.52	0.84	0.87	0.91
Interacting with practicing physicians	0.52	0.71	0.69	0.34	0.84	0.27	0.74	0.65	0.66
Feeling the camaraderie of my peers	0.40	0.80	0.83	1.00	0.05	0.28	0.84	0.77	0.85
Earning a salary for practicing medicine	0.88	0.50	0.49	0.88	0.34	0.77	0.47	0.44	0.49
Being responsible for decisions regarding patients	0.16	0.71	0.75	0.38	0.57	0.61	0.72	0.75	0.78
Feeling competent about patient care	***0.00***	0.83	0.91	0.28	***0.04***	1.00	0.88	0.87	0.93
Contributing to the teaching program of students	0.88	0.63	0.62	0.27	0.76	0.36	0.58	0.65	0.66
Being legally responsible for patient care	0.78	0.08	0.07	0.28	0.31	0.06	0.12	0.05	0.07
Being finished with classroom duties	0.92	0.18	0.18	1.00	0.80	0.85	0.16	0.18	0.17
Knowing people look up to me	0.14	0.20	0.25	1.00	0.25	0.46	0.25	0.20	0.24
Establishing good communication with patients	0.74	0.74	0.75	0.49	0.56	0.29	0.72	0.79	0.76
Exercising good clinical judgment	0.19	0.83	0.87	***0.03***	0.89	***0.04***	0.75	0.87	0.88
Learning from interesting clinical cases	0.53	0.79	0.81	1.00	0.81	0.71	0.79	0.81	0.80
Having one less year to train	0.36	0.27	0.24	1.00	0.75	0.87	0.26	0.28	0.26

Meeting unrealistic expectations of faculty	0.94	0.33	0.33	***0.01***	0.92	***0.02***	0.51	0.33	0.33
Having no free time	0.25	0.63	0.67	0.72	0.13	0.19	0.79	0.69	0.76
Seeing patients die	***0.00***	0.31	0.21	0.12	***0.02***	1.00	0.33	0.33	0.23
Coping with patients who have self-destructive diseases	***0.02***	0.50	0.41	0.76	0.10	0.55	0.53	0.58	0.50
Worrying about money decisions	0.51	0.50	0.47	0.18	0.63	0.30	0.56	0.48	0.46
Being required to make decisions beyond my competence	0.35	0.24	0.27	0.87	0.23	0.60	0.26	0.23	0.28
Getting too little sleep	***0.01***	0.55	0.65	0.61	***0.02***	0.44	0.70	0.63	0.74
Having to see too many patients in too little time	0.88	0.43	0.44	***0.01***	1.00	***0.01***	0.68	0.48	0.49
Dealing with angry, unresponsive, disrespectful patients	0.83	0.46	0.47	0.45	0.63	0.24	0.60	0.50	0.53
Missing my family/spouse	0.46	0.56	0.59	0.05	0.27	***0.01***	0.79	0.60	0.65
Feeling uncertain about my medical decisions	0.77	0.41	0.39	1.00	0.92	0.88	0.40	0.39	0.40
Endless gray clouds; never seeing the sun rise or set	0.40	0.24	0.27	0.20	0.19	***0.03***	0.39	0.24	0.30
Coping with different advice/opinion/instructions of consulting staff	***0.03***	0.31	0.39	0.36	0.27	0.88	0.32	0.33	0.39
Feeling anxious about my ability to perform physician skills	0.06	0.32	0.39	0.45	***0.03***	0.53	0.37	0.32	0.43
Being criticized or put down by consulting staff	0.86	0.22	0.23	***0.03***	0.82	***0.02***	0.39	0.23	0.24
Seeing the same kinds of patients over and over	1.00	0.08	0.08	0.64	0.40	0.30	0.12	0.08	0.10
Spending too much time with paperwork	0.24	0.60	0.56	0.12	0.92	0.06	0.72	0.60	0.60

The p-values were computed by the Fisher's exact test followed by the corresponding proportions of "yes" responses to each question. The p-values in italics are significant at 5% level. For details of the column headings, see Figure 1 caption.
